# Wild Micromammals as Bioindicators of Antibiotic Resistance in Ecopathology in Northern Italy

**DOI:** 10.3390/ani10071184

**Published:** 2020-07-13

**Authors:** Giovanna Zanardi, Tiziano Iemmi, Costanza Spadini, Simone Taddei, Sandro Cavirani, Clotilde Silvia Cabassi

**Affiliations:** Department of Veterinary Science, University of Parma, via del Taglio 10, 43126 Parma, Italy; giovanna.zanardi.vet@gmail.com (G.Z.); tiziano.iemmi@unipr.it (T.I.); costanza.spadini@unipr.it (C.S.); simone.taddei@unipr.it (S.T.); sandro.cavirani@unipr.it (S.C.)

**Keywords:** antibiotic-resistance, bioindicators, ecopathology, wild micromammals

## Abstract

**Simple Summary:**

In recent years, animal and human health have been linked in a “One Health” approach. Wildlife could act as a reservoir for different antibiotic-resistant pathogens, representing an issue for human and domestic animal health. The aim of this study was to evaluate the presence and circulation of antimicrobial-resistant bacterial species in wild micromammals in the province of Parma, Northern Italy. Multi-drug-resistant strains and a high prevalence of resistance to critically important antibiotics were detected. Furthermore, resistance to commonly used antibiotics was detected in a large percentage of isolates. Considering that micromammals are good bioindicators, obtained results highlighted a high prevalence of strains resistant to antimicrobials of critical importance for human and animals in the investigated areas, thus representing a public health hazard.

**Abstract:**

Antimicrobial resistance (AMR) is an increasing threat to human health and an important issue also in the natural environment. For this study, an ecopathological approach was applied to the monitoring of the antimicrobial resistance in the province of Parma, Northern Italy. Fourteen monitoring sites and seventy-four faecal samples from four species of wild micromammals (*Apodemus sylvaticus, Microtus savii, Mus domesticus* and *Suncus etruscus*) were collected. Samples were subjected to bacteriological examination and antimicrobial susceptibility testing. Antibiotics belonging to 13 different antibiotic classes were tested. Collected data showed a prevalence of multi-drug resistant (MDR) strains of 55.13% and significant differences in the prevalence of MDR strains among the different micromammal species, while sex, age and anthropization level did not significantly affected MDR strains prevalence. Moreover, a high prevalence of bacterial strains resistant to colistin (95%), gentamicin (87%) and amikacin (83%) was observed. To our knowledge, this is the first report on antibiotic resistance in wild micromammals in the province of Parma.

## 1. Introduction

The global expansion of human activities is causing increased anthropogenic pressure on the environment, leading to changes of wildlife–livestock–human interfaces and favouring the emergence and re-emergence of infectious diseases [1]. The management of wildlife health is closely linked to the human and veterinary public health in a “One Health” approach, and one of the main aims is also to monitor and counteract the spread of antimicrobial resistance (AMR) in natural environment [2]. Bacterial expression of acquired resistance is promoted by a massive use of antimicrobial agents, both in human and veterinary medicine [3]. The presence of AMR strains in natural environment is associated with different mechanisms: natural production of antimicrobial molecules from bacteria and fungi [4], horizontal transmission of resistance determinants [5], and as a consequence of the presence of anthropogenic antibiotics, which could be considered as environmental pollutants [6]. Restrictions on antimicrobial use could enhance their removal from natural ecosystem [7]. Conversely, once antibiotic resistance genes are present in gene-transfer platforms, the probability for their maintenance in natural ecosystems could be high. For this reason, antibiotic resistance genes are considered to be pollutants themselves [6].

Usually, wildlife is not directly exposed to clinical antimicrobial agents, but can acquire antimicrobial-resistant bacteria through contact with humans, animals and the environment. Water polluted with faeces appears to be the most significant vector of contamination [5,8]. This condition promotes the emergence of AMR, and wildlife commensal bacterial strains may become reservoirs of virulent and resistant genes [9,10]. It has now been established that AMR bacteria are ubiquitous in natural ecosystems and are widespread in wild populations [11,12]. This strongly suggests that the widespread resistance found in bacterial populations may be caused by human activities. Conversely, it has been reported that wild population that has never been exposed to humans is free of resistance to antibiotics [13]. To provide a broader picture of AMR in wildlife, prevalence studies across different habitats, combined with other spatial data (e.g., the presence of fosterage and human influence), should be encouraged [8,14,15].

Wild animals may act as bioindicators of AMR contamination [5,8,16]. To understand their potential as bioindicators of AMR contamination, it is essential to choose animal species characterized by favourable ecological traits, with the aim of detect the origin of the habitat contamination [17,18,19]. The knowledge of species-specific characteristics makes it possible to understand the role of these species in the evolution, maintenance and dispersion of AMR bacteria [8]. Bioindicators include biological processes, species, or communities that are used to assess the quality of the environment over time and, from a management perspective, give information of what is and is not biologically sustainable [19]. Bioindicators are valued through changes in their individual fitness, population density, community composition and ecosystem processes. The density of the bioindicators must be adequate and stable over time and the response must be measurable [19].

The main AMR bioindicators among wild species include birds of prey, waterfowl and wild small mammals [20,21,22]. Small mammals interact frequently with human and agricultural waste materials, and thus they are potentially exposed to anthropogenic sources of AMR in the environment [5,22]. Their ubiquitous nature, small home range size and often generalist diets makes them suitable for detecting previously unstudied fine-scale variations in AMR distribution patterns [22,23]. Furthermore, different wild small mammal species can show different ways of spread AMR in the same environment, on the basis of the different ecology and biology of the species [24]. AMR Gram-negative bacteria persist in the gut of humans and animals, especially those treated with antibiotics and, through diffusion in the environment, water and food, can contaminate both humans and animals. The control of this diffusion is crucial [16].

The aim of this study was to investigate the prevalence of Gram-negative AMR strains in wild small mammal populations in different geographic sites in the province of Parma, in Northern Italy.

To our knowledge, this is the first report on antibiotic resistance in wild micromammals in the investigated area.

## 2. Materials and Methods 

### 2.1. Animals and Study Areas

Fourteen sampling areas, homogeneously distributed within the province of Parma (Northern Italy), were selected. Sampling sites were selected within sampling areas on the basis of the potential presence of wild small mammals. Sampling areas were identified with letters from “A” to “N”. Catches were made between October 2016 and November 2017. Catches in a free environment were conducted from August 21, 2017, after obtaining catching authorization from the Agenzia Regionale per la Prevenzione, l’Ambiente e l’Energia (ARPAE) of the Emilia-Romagna region. The capture of small mammals was performed following the indications reported by the executive resolution n. DET-AMB-2017-4392 of 17/08/21. The purpose of the study, municipal territories of the capture sites, species of interest, type of sampling, type of traps, distance of the traps, sampling and animal releasing protocols are indicated in the resolution. Briefly, 74 wild micromammals were captured and identified by a letter indicating the site of capture, followed by a serial number indicating the chronology of capture (e.g., A1, A2, …, C1, C2, etc.). The considered species were *Apodemus sylvaticus* (n = 31)*, Microtus savii* (n = 10)*, Mus domesticus* (n = 29)*, Suncus etruscus* (n = 4). For each animal capture site, trap type, date and time of capture, species, sex, weight and age, were registered. Animals were divided into juveniles and adults based on the evaluation of the development of secondary sexual organs. For analytical evaluation, three parameters were considered: altitude, urbanization and domestic animal presence. Sampling sites were georeferenced by GPS (Table 1).

#### 2.1.1. Altitude

The altitude of the sampling sites ranged from 26 to 962 m above sea level. Three altitude ranges were defined: 0–99, 100–499 and over 500 m above sea level (Table 1).

#### 2.1.2. Urbanization and Domestic Animal Presence

Sites were considered far from urbanization areas and domestic animals if trap distance from human activities was at least 250 m. This distance was evaluated based on the home range of sampled species [25,26,27,28,29] (Table 1).

### 2.2. Sampling

Sampling procedures were carried out with particular care for proper animal handling. Each subject was morphologically evaluated, and a rectal swab was taken. Animals were manually restrained for the minimum time necessary to perform sampling procedures and then immediately released. Swabs were immediately placed into AMIES transport medium and identified with a serial number. Samples were refrigerated at 4 °C in a portable fridge and delivered within 24 h to the laboratory for bacteriological investigation.

### 2.3. Cultural Examination

Rectal swabs from healthy wild small mammals were plated on solid plates of Agar MacConkey to evaluate the presence of bacteria belonging to the *Enterobacteriaceae* family. Plates were incubated at 37 °C for 24 h in aerobic atmosphere. Identification of bacterial isolates was based on growth and colony characteristics, Gram staining, cellular morphology, catalase and oxidase reactions and Gram-negative aerobic strains species identification was carried out by API 20E (bioMérieux, France) [30].

### 2.4. Antimicrobial Susceptibility

Antimicrobial susceptibility test was performed by agar disk diffusion method, according to CLSI guidelines [31]. A panel of 17 antimicrobial molecules were tested, following CLSI guidelines for *Enterobacteriaceae*. Selected antibiotics are subdivided into categories and are listed in Table 2. Briefly, antibiotic disks were placed on the surface of Antibiotic Medium 1 Agar plates (Difco, Sparks, USA) inoculated with the bacterial strain under investigation. Plates were then incubated at 37 °C in aerobic conditions for 24 h. After incubation, the diameters of growth inhibition zones were measured and compared with those reported by CLSI guidelines to determine bacterial susceptibility or resistance [31,32].

Each isolate was defined as sensible (S), intermediate (I) or resistant (R) to each tested antibiotic. Each strain was defined, following the guidelines reported by Magiorakos et al. (2012), as multi-drug resistant (MDR), extensively drug resistant (XDR), pan-drug resistant (PDR), or non-MDR. Colistin is reported in the list of antibiotics to be tested in presence of MDR, XDR and PDR organisms [33]. Colistin breakpoints reported in the section “Non-Fastidious Bacteria” of the CLSI guidelines were used [32]. Referring to Galani et al. [34], evaluation of susceptibility to colistin for intermediate strains was performed by MIC test, following CLSI guidelines [32].

### 2.5. Statistical Analysis

Statistical significance was evaluated by chi-squared test with Yates correction when the total number of events was greater than or equal to 40 or by Fisher’s exact test when the total number of events was lower than 40. A 0.05 level of statistical significance (*p*-value) was chosen. In the statistical calculation of resistance to individual antibiotics, bacteria intrinsically resistant to those antibiotics were excluded [33,35].

## 3. Results

Seventy-eight bacterial strains were isolated from rectal swabs. Isolates belonged to 13 different species: *Escherichia coli* (53), *Enterobacter cloacae* (4), *Pantoea* spp. (4), *Hafnia alvei* (3), *Raoultella ornithinolitica* (3), *Cronobacter* spp. (2), *Enterobacter amnigenus* (1), *Escherichia vulneris* (1), *Klebsiella pneumoniae* (1), *Serratia fonticola* (1), *Serratia liquefaciens* (1), *Pseudomonas oryzihabitans* (1).

Antibiotic sensitivity was evaluated for each strain and results are showed in Table 2 [33].

The percentage of resistance of *E. coli* strains, the most represented bacterial species on the total of the collected samples (53 of 78), was highlighted (Table 3).

Among the 78 isolates, 35 were non-MDR, 43 MDR, and there were no XDR or PDR. The percentage of MDR on total strains was 55.13%. For each sampling site the percentage of MDR isolates was reported in Table 4.

Except for *E. coli,* the number of isolates was too low for statistical analysis. Among the 53 *E. coli* isolates, 22 were MDR (41.51%). Out of four *Pantoea* sp isolates, only one strain was MDR (25%). All the other bacterial isolates were MDR (100%).

### 3.1. Altitude

Regarding the altitude, no statistically significant differences were found in the percentage of MDR strains at different altitudes; in particular, *p* = 0.789 was found for the comparisons of zone I (53.85% of MDR) and II (46.67% of MDR); *p* = 0.474 for I and III (68.18% of MDR); *p* = 0.207 for II and III.

### 3.2. Species

The percentages of MDR isolates were 100% for *S. etruscus,* 78.57% for *M. savii*, 50% for *M. domesticus* and 37.04% for *A. sylvaticus*. Statistical analysis of association between MDR strains and small mammal species revealed a statistically significant difference between *M. domesticus* and *S. etruscus* (*p* = 0.028), *A. sylvaticus* and *S. etruscus* (*p* = 0.007) and *A. sylvaticus* and *M. savii* (*p* = 0.028). No statistically significant differences were found between *M. domesticus* and *A. sylvaticus* (*p* = 0.473), *M. domesticus* and *M. savii* (*p* = 0.143) and *S. etruscus* and *M. savii* (*p* = 0.521).

### 3.3. Age and Sex

Of the 74 animals captured, age was registered for only 67 and sex for 57. Comparison of young animals (on 11 isolates, 4 were MDR with a percentage of 36.36%) with adults (of 67 isolates, 39 were MDR, with a percentage of 58.21%) showed no significant difference (*p* = 0.306) regarding MDR prevalence. No statistically significant difference in MDR prevalence was also detected between males (of 31 isolates, 18 were MDR, with a percentage of 58.06%) and females (of 26 isolates, 14 were MDR, with a percentage of 53.85%) (*p* = 0.959).

### 3.4. Urbanization Levels

Overall, the percentage of MDR strains among isolates from animals captured in areas far from human activities (250 m) (E, F, L, and M areas; 19 total isolates and 7 MDR) was 36.84%. The difference with the other areas (A, B, C, D, H, I, J, K and N; 59 total isolates and 36 MDR), where the percentage was 61.02%, was not statistically significant (*p* = 0.115).

### 3.5. Antibiotics

All strains showed resistance to one or more of the tested antibiotics. Moreover, no antibiotic was effective on all of the 78 tested strains. Resistance percentage to the different antibiotics ranged between 4% (3/78) for imipenem to 95% (74/78) for colistin. Percentages of resistant strains are reported in Table 2.

Due to the slow diffusion of polymyxins in agar, the only validated test method for colistin is the evaluation of the “Minimal Inhibitory Concentration” (MIC) [31,32]. Reference resistance limits of colistin for *Enterobacteriaceae* are reported by CLSI and EUCAST in the section “Non-Fastidious Bacteria” under the heading “*Escherichia coli* ATCC 25922“ [34]. In our study, four samples were sensitive to colistin (Table 2 and Table 3).

## 4. Discussion

From a perspective of “One Heath” worldwide policy, ecopathology was created with the aim of managing infectious disease, both of animals and humans, with an epidemiologic, ecologic and environmental approach [15]. In ecopathological studies, measuring each potentially relevant aspect within an ecosystem is not possible [36]. To establish the level of environmental antibiotic contamination in the investigated area, representative data are required. Bioindicators can be used to reduce the numbers of components that must be investigated and monitored [15,36]. In the present study, the disk diffusion method was chosen for the evaluation of antibiotic resistance of bacterial isolates, in order to evaluate many samples and antibiotics to set up a monitoring plan. A single species should not be expected to act as an indicator for an entire ecosystem unless two species occupy the same niche [37,38]. Each species represents a different ecological niche and manifests different sensitivity to environmental changes. Therefore, the highest number of species living in a specific area should be monitored in order to limit interpretation errors [36]. For this reason, we considered four species of autochthonous small mammals with different eco-ethologic characteristics.

Monitoring the spread of antibiotic-resistant bacterial strains in wild small mammal populations over time could be a useful method for evaluating the persistence of environmental antimicrobial resistance [11,39]. Therefore, the present study could be repeated over time to assess the trend of antibiotic-resistance through spatial and temporal mapping of antibiotic resistance in the area of concern. This method could allow evaluation of the effectiveness of control measures and to identify the correct management of this increasingly looming problem.

We found statistically significant differences in the prevalence of MDR bacterial isolates between micromammal species with wide eco-ethologic differences. For example, *S. etruscus* is a predator of arthropods and invertebrate and has a large home range, while *M. domesticus* and *A. sylvaticus* are omnivorous and with a smaller home range [25,26,27,28,29]. The difference between *A. sylvaticus* and *M. savii* was statistically significant. However, this result could be influenced by the area of sampling: all *M. savii* isolates were derived from subjects from a single area in the proximity of human activities (Site A), while most of *A. sylvaticus* (19/27 isolates) were derived from areas far from human activities.

The interspecific differences can also be useful for monitoring the trend of antibiotic resistant strains spreading. In fact, the behaviour of subjects of the same species can be different according to age and gender. Young subjects are thought to be less exposed to antibiotics than adults during their lifetime. Consequently, a lower prevalence of MDR isolates in younger compared to older animals could be expected. However, our results showed no significant difference related to the age of the animals.

Regarding sex, usually females have a slightly reduced home range, limited to the surroundings of the den, compared to the males [25,26,27,28,29]. However, no statistically significant difference was observed related to sex. The lack of statistically significant differences in the spread of MDR strains between young and adults and between males and females may be due to the wide diffusion of antibiotic resistance in the environments and the low home range of these micromammals.

Antimicrobial resistance is related to the frequency of resistance genes in bacterial population and the presence of MDR isolates is a function of this spread through the environment, in wild and domestic populations [3].

Our results were similar to those found in the northwest of England [11], where the resistance to antibiotics is widespread in some wild populations, even though it is supposed that these populations have never been exposed to antibiotics. We found similar high percentages of MDR strains, both in areas far from human activities, in which the spread of antibiotics in the environment could be expected lower, as well as in areas with higher anthropization. Moreover, the lack of significant difference between altitude zones suggests that antibiotic resistance is largely widespread on all the investigated areas. This could be linked to a significant human presence, as well as the presence of intensive agricultural activities, in the considered areas [13].

The prevalence of colistin, gentamicin and amikacin resistant strains was high. Imipenem showed the lowest spectrum of resistance. No antibiotic was effective against all isolated strains.

As reported by the European Medicines Agency (EMA), colistin was abandoned several years after its first use due to harmful antibiotic accumulation. Recently, it has been reintroduced in human therapies as it represents the antibiotic of choice in serious Gram-negative infections not responsive to others antibiotics such as carbapenems [40]. The development of new resistance genes, such as *mcr-1*, transmitted by plasmids, has placed global attention on colistin, which has become the emblem of the global AMR issue [41]. This could explain the colistin resistance results found in this study. In Italy, since 2016, following the EMA opinion, attempts to counteract the increase in colistin resistance were carried out by limiting the use of colistin in livestock [40].

Correct considerations on resistance to other antimicrobial classes also should not disregard the use of antimicrobial agents in human and veterinary medicine throughout the years. Resistance against penicillins and first-generation cephalosporins was the first to emerge and in the 2000s resistance to aminoglycosides, third-generation cephalosporins and quinolones was reported [16]. The antibiotic-producing bacteria are widespread in the environment and due to the large-scale mixing of these environmental bacteria with exogenous bacteria from anthropogenic sources they provide the ideal ecological conditions for the emergence of new resistant strains [16]. Therefore, we expected to find a significant number of bacterial strains resistant to the tested antibiotics due to the high density of human activities in the considered territory.

Our results showed a high resistance level to some of the tested antibiotics. In particular, colistin resistance was considerable. Colistin and aminoglycoside (gentamicin and amikacin) resistance are critical for human health, as they are widely used both in human and veterinary medicine. The data obtained are representative of the environmental and temporal situation considered and are a starting point for future research. The elaboration of a protocol which takes into consideration natural and human factors within a limited geographical area could be useful both for the organization of environmental surveillance plans for different territorial typologies and to assess the impact of specific human activities, such as livestock farms, on the surrounding environment.

### Limits of the Study

Possible limits of the present study could be related to the variability of antibiotic resistance prevalence depending on different elements such as the eco-ethology of the different animal species and, within the same species, in relation to changes in environmental conditions, trophic sources and reproductive state [24]. Increasing the sample size, the number of sampling sites and repeating the survey over time, also in consideration of possible seasonal variations, could allow to obtain further indications regarding the monitoring of resistance.

## 5. Conclusions

Antibiotic resistance is a crucial concern on a global level. Political efforts at an international level aim to identify new control methods and, above all, the prevention and monitoring of emerging antibiotic resistance. Attention to human health cannot ignore the issue of animal and environmental health [15]. Therefore, the monitoring should be done at all three levels, and data should be collected and compared over time to assess the changes that occur in these macrosystems. Wild micromammals represent both a reservoir and a valuable bioindicator of antibiotic resistance. They can spread antimicrobial resistance, with consequent impact on human and veterinary medicine [24].

Obtained results showed significant differences in the prevalence of MDR strains among the different animal species, while sex, age and environmental anthropization level do not significantly affected MDR strains prevalence. Further studies to define more precisely the levels of environmental resistance are required. However, a very interesting finding was the high prevalence of strains resistant to colistin, amikacin and gentamicin. Resistance to the aforementioned antibiotics was widespread and this could represent a public health hazard in the considered areas.

## Figures and Tables

**Table 1:** Sites of sampling characterization: site name (A–N); altitude; zones of altitude (three ranges, zone 1: between 0 and 99 m; zone 2: between 100 and 499 m; zone 3: over 500 m); urbanization and domestic animal presence (under 250 m from human activity: yes; over 250 m: no); latitude and longitude (GPS georeferentiation).

Site	Altitude (m)	Zones of Altitude	Urbanization and Domestic Animal Presence	Latitude	Longitude
A	43	1	Yes	44°50′6.95′′ N	10°17′58.15′′ E
B	154	2	Yes	44°38′59.80′′ N	10°23′5.81′′ E
C	52	1	Yes	44°50′13.31′′ N	10°19′9.19′′ E
D	42	1	Yes	44°51′21.76′′ N	10°19′54.62′′ E
E	145	2	No	44°38′7.51′′ N	10°24′55.67′′ E
F	26	1	No	44°55′15.36′′ N	10°27′10.13′′ E
H	219	2	Yes	44°39′17.97′′ N	10°16′39.99′′ E
I	744	3	Yes	44°30′29.58′′ N	9°55′14.16′′ E
K	623	3	Yes	44°34′2.15′′ N	9°53′57.57′′ E
L	962	3	No	44°29′29.92′′ N	10°16′51.77′′ E
M	421	2	No	44°35′32.25′′ N	11° 6′42.17′′ E
N	164	2	Yes	44°40′18.31′′ N	10°19′53.94′′ E

**Table 2:** Resistant (R), intermediate (I) and sensible (S) strains on total isolates and percentages of resistant strains for each tested antibiotic. Intermediate (I) strains were considered resistant for percentages of resistance calculation, except for colistin, that intermediate strains at disk diffusion test resulted sensible at MIC test (4/78).

Antibiotics Classes	Antibiotics	I	R	S	Tot.	Resistance (%)
Aminoglicosides	Amikacin	12	53	13	78	83%
	Gentamicin	4	64	10	78	87%
Penicillins and β-lattamase inhibitors	Amoxicillin + Clavulanic Acid	4	7	59	70	16%
Penicillins	Ampicillin	5	15	49	69	29%
Monobactams	Aztreonam	2	10	66	78	15%
Cephalosporin I° generation	Cephazolin	3	22	45	70	36%
Cephalosporin III° generation	Cefotaxime	5	9	64	78	18%
	Ceftazidime	4	6	68	78	13%
	Ceftriaxone	6	3	69	78	12%
Fluoroquinolones	Ciprofloxacin	8	3	67	78	14%
	Enrofloxacin	6	6	66	78	15%
Phenicols	Chloramphenicol	2	5	71	78	9%
Polimixins	Colistin	0	74	4	78	95%
Carbapenems	Imipenem	1	2	75	78	4%
Penicillin Anti-Pseudomonas	Piperacillin + Tazobactam	4	3	71	78	9%
Tetracyclines	Tetracycline	3	11	64	78	18%
Folate inhibitors	Trimethoprim+ Sulfamethoxazole	7	14	57	78	27%

**Table 3:** Resistance percentages of *E. coli* strains. Intermediate (I) strains were considered resistant for percentages of resistance calculations.

Antibiotics	I	R	S	Tot.	Resistance (%)
Amikacin	6	39	8	53	85%
Gentamicin	1	44	8	53	85%
Amoxicillin + Clavulanic Acid	2	4	47	53	11%
Ampicillin	2	10	41	53	23%
Aztreonam	1	7	45	53	15%
Cephazolin	2	10	41	53	23%
Cefotaxime	1	3	49	53	8%
Ceftazidime	1	1	51	53	4%
Ceftriaxone	2	1	50	53	6%
Ciprofloxacin	3	2	48	53	9%
Enrofloxacin	2	2	49	53	8%
Chloramphenicol	1	3	49	53	8%
Colistin	0	50	3	53	94%
Imipenem	1	2	50	53	6%
Piperacillin + Tazobactam	1	0	52	53	2%
Tetracycline	1	9	43	53	19%
Trimethoprim+ Sulfamethoxazole	2	6	45	53	15%

**Table 4:** MDR percentage in relation to sampling sites.

Site	Isolates	MDR	%
A	16	11	68.75%
B	7	4	57.14%
C	5	0	0%
D	4	2	50.00%
E	10	1	10.00%
F	1	1	100%
H	4	1	25.00%
I	11	7	63.64%
K	6	5	83.33%
L	5	3	60.00%
M	3	2	66.67%
N	6	6	100%
